# Can Molecular Breast Imaging With Tc-99m Sestamibi Safely Rule Out Malignancy in Pathologic Nipple Discharge?

**DOI:** 10.1097/RLU.0000000000005851

**Published:** 2025-04-30

**Authors:** Sofia C. Vaz, Christinne L.S. Corion, Jelle Goeman, Anneke M. Zeillemaker, Rachel Hezemans, Lioe-Fee de Geus-Oei, Lenka M. Pereira Arias-Bouda

**Affiliations:** * Nuclear Medicine-Radiopharmacology, Champalimaud Clinical Center, Champalimaud Foundation, Lisbon, Portugal; † Department of Radiology, Leiden University Medical Center, Leiden; ‡ Department of Surgery, Haaglanden Medisch Centrum, Den Haag; § Department of Biomedical Data Sciences, Leiden University Medical Center, Leiden; ∥ Department of Surgery, Alrijne Hospital; ¶ Department of Nuclear Medicine, Alrijne Hospital, Leiderdorp; # Biomedical Photonic Imaging Group, University of Twente, Enschede; ** Department of Radiation Science & Technology, Delft University of Technology, Delft

**Keywords:** molecular breast imaging, breast cancer, nipple discharge, breast imaging, Tc-99m sestamibi, gamma-camara scintigraphy

## Abstract

**Purpose:**

Nipple discharge is the third most common breast-related complaint. It is recommended to exclude malignancy in pathologic nipple discharge (PND). Mammography and ultrasound are the first-line conventional diagnostic (CD) imaging. Although magnetic resonance is often used as a complementary modality, molecular breast imaging (MBI) with Tc-99m sestamibi may be a suitable alternative. Considering the lack of information on this subject and its clinical importance, this study aimed to evaluate the role of MBI in ruling out malignancy in patients with PND and negative/indeterminate CD.

**Patients and Methods:**

Retrospective cohort single-center study including all patients with PND evaluated by CD and MBI between 2012 and 2020. Pathology was considered the gold standard. Follow-up was used when pathology was not available.

**Results:**

Of the 96 cases of PND included, 78 were benign, and 18 (20%) corresponded to breast cancer (BC). Although CD and MBI were concordant in the BIRADS classification in 81% (78/96), half of BC were detected by MBI only. BC was located directly behind the nipple in a minority of patients (11%), meaning that MBI could significantly prevent futile central ductal excision. MBI presented higher sensitivity (83% vs. 33%) and negative predictive value (96% vs. 86%) than CD alone, with similar specificity (89% vs. 92%) and positive predictive value (63% vs. 50%). The area under the curve of MBI and CD was 0.86 (*P*-value<0.001 [95% CI: 0.75–0.97]) and 0.63 (*P*-value=0.091 [95% CI: 0.47-0.79]), respectively.

**Conclusions:**

MBI showed good diagnostic accuracy for detecting BC in patients with PND with negative/indeterminate findings on CD imaging.

Nipple discharge is the third most common breast-related complaint, after pain and palpable breast mass. Nipple discharge can be characterized as physiological or pathologic. Physiological nipple discharge is often provoked, originating from multiple ducts, bilateral, and may present with white, green, or yellow colors.^1^ It is rarely associated with cancer (0.3%).^2^ On the other hand, pathologic nipple discharge (PND) has at least one of the following features: spontaneous, unilateral secretion, discharging from a single duct, being persistent and sometimes serous or bloody. Generally, PND has a benign cause, such as intraductal papilloma/papillomatosis (35%–48%) and duct ectasia (17%–36%).^3–5^ The rate of malignancy in PND is around 11%–16%.^3,4,6–8^

The clinical workup in patients with nipple discharge should include a detailed clinical history and physical examination that directs the need for any type of breast imaging.^9^ Usually both mammography (MG) and ultrasound (US) are used to rule out an underlying malignancy. Lesions are often retroareolar, small, completely intraductal, and lack calcification, impeding the mammographic detection of pathologies in these patients, leading to decreased sensitivity, around 20-25%, compared with patients presenting with a palpable mass.^1,10–12^ The sensitivity and specificity of US for detection of malignancy in patients presenting with PND ranges from 63%-100% and 73%–84%, respectively.^13^ Some practices also perform cytologic examination, ductography, and magnetic resonance imaging (MRI).^14,15^ The added value of MRI is, however, uncertain in patients with PND with no signs of malignancy on conventional imaging.^16–18^

The uncertain diagnosis and the possibility of a malignant lesion lead to surgical interventions such as duct excision or microdochectomy. These procedures have associated risks, such as wound infections and scarring, and can lead to future breastfeeding difficulties in fertile women. Some patients report discharge even after microdochectomy because some lesions are not located behind the nipple and can be missed. Keeping in mind that malignancy is only found in 11%–16% of patients, futile surgical intervention may be performed in up to 80%–90% of patients. Due to the limited sensitivity and specificity of conventional radiologic imaging in detecting malignant lesions in patients with nipple discharge, there is a clinical need for other techniques. Despite MRI being the most commonly used adjunct modality,^19^ molecular breast imaging (MBI) seems to be a good alternative because it is not limited by structural modifications, breast densities, breast/metal implants, or other devices. Furthermore, MBI is well tolerated by patients and, therefore, it is more suitable for patients with obesity or claustrophobia. It has few contraindications (eg, pregnancy and in the rare case of allergic reaction to radiopharmaceutical), it is less expensive with a greater cost-effectiveness outcome and has a faster interpretation learning curve.^20,21^

MBI can be performed either with dedicated gamma-camera equipment that detects single-photon emitting radiotracers (such as sestamibi labeled with technetium 99 metastable - Tc-99m sestamibi) or with dedicated coincidence detection systems that detect positron-emitting radiotracers [such as 2-deoxy-2-(^18^F)fluoro-d-glucose labeled with fluoride 18—^18^F-FDG], which are named as dedicated breast PET (DbPET) or positron emission mammography (PEM).^22^ MBI with Tc-99m sestamibi has been proven a valuable tool as an adjunct imaging modality. Tc-99m sestamibi is Food and Drug Administration (FDA)–approved as a second-line diagnostic tracer after mammography to study breast lesions and European Medicines Agency (EMA)–approved for identification of breast cancer when mammography is equivocal, inadequate, or indeterminate.^22^ The uptake of Tc-99m sestamibi in breast cancer cells is based on increased mitochondrial activity and increased vascularity.^23^ Breast-dedicated gamma-cameras can detect small breast lesions (≤1 cm) at a relatively low radiation dose. MBI also offers the option for MBI-guided biopsy.^22^

The SNMMI Procedure Standard/EANM Practice Guideline for Molecular Breast Imaging with Dedicated gamma-Cameras published in 2022 state that the common indications for MBI are the evaluation of indeterminate imaging findings, local staging, monitoring response to neoadjuvant therapy, screening, surveillance for breast cancer recurrence and problem-solving in case of dense breasts, implants, free silicone, claustrophobia, or when breast MRI is contraindicated or unavailable.^22^ Several studies showed similar sensitivity between MBI and MRI (89%–97%), but higher specificity rates of MBI compared with MRI (77%–89% vs. 40%–68%).^24,25^

In a retrospective work performed in our institution, we evaluated MBI conducted in 226 patients with equivocal breast abnormalities after conventional diagnostic imaging with mammography and ultrasound (CD).^26^ We found discordant findings between MBI and CD imaging, enabling correct adjustment of treatment in 20% of patients, mostly due to detection of malignant lesions by MBI not identified by other modalities.^26^ We also verified that in the subgroup of patients with nipple discharge, MBI correctly adjusted imaging classification in 21%, in particular, by identifying breast cancer in 19% of patients (none had been depicted on mammography and ultrasound).

Considering the limited knowledge of breast imaging in nipple discharge and the clinical importance of accurately differentiating benign from malignant disease in a noninvasive way in the case of PND, the current study was conducted.

## METHODS

### Patient Selection

**FIGURE 1:**
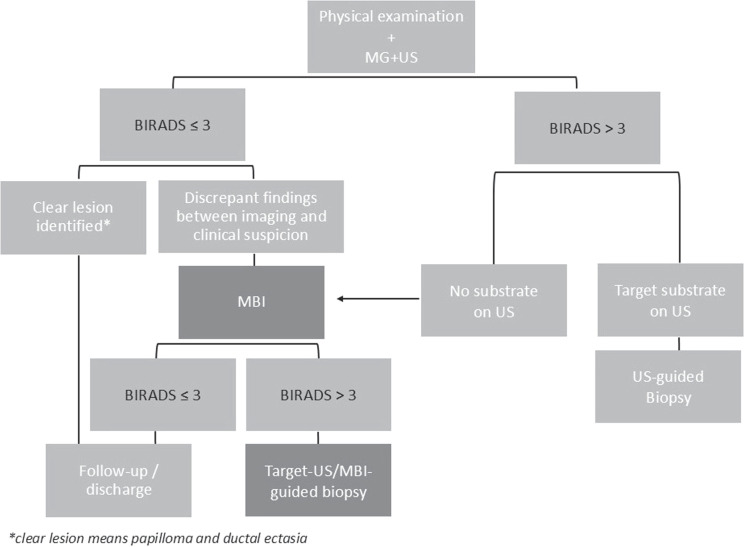
Flowchart representing the investigation procedure of PND (pathologic nipple discharge).

This retrospective cohort single-center study included all women who presented with PND between April 2012 and September 2020 and who underwent MBI with Tc-99m sestamibi after CD imaging in the Alrijne Hospital in Leiderdorp, the Netherlands. CD imaging was performed with MG and US. If the lesion was scored as Breast Imaging Reporting and Data System (BIRADS) ≤3 and a clear lesion, such as papilloma and ductal ectasia, was the most probable diagnosis, patients were discharged or kept in follow-up, if needed. On the other hand, if the lesion was scored as BIRADS>3, the diagnostic workup was completed with fine needle biopsy (FNB)/ core needle biopsy (CNB) whenever possible (Fig. 1). In case of discrepant clinical and imaging findings, or when no substrate was found on the US for biopsy, patients underwent MBI. MBI-guided biopsy was performed when the lesion was classified as BIRADS>3 on MBI. The patient was discharged or kept in follow-up when the lesion was classified as BIRADS≤2 or 3, respectively (Fig. 1). Exclusion criteria included histopathologically confirmed BC before MBI and incomplete medical files. This study was approved by the local regulatory authorities.

### Imaging Procedures

MG imaging of the breast consisted of routine digital craniocaudal (CC) and mediolateral oblique (MLO) views (Siemens Inspiration Mammomat). Whenever necessary, target US was performed (Philips Affiniti 70 G Linear transducer L 12-5). MG and US were interpreted by a radiologist according to BIRADS^27–29^ (Table 1).

**TABLE 1:** Mammography and MBI BI-RADS Assessment Criteria^29,30^

BIRADS category		Interpretation	Recommendation
0	Incomplete	Not possible	Additional imaging or comparison with previous images
1	Negative	No lesion found	Routine follow-up
2	Benign	No malignant features	Routine follow-up
3	Probably benign	Very low probability of malignancy (<2%)	Short interval follow-up (6 mo)/follow-up MBI examination is recommended in 6 mo if mammography and ultrasound are negative
4	Suspicious/intermediate	2%–94% probability of malignancy	Biopsy should be considered/is recommended
4a	Low suspicion	Low suspicion for malignancy (2%–9%)	
4b	Moderate/intermediate suspicion	Moderate/intermediate suspicion for malignancy (10%–49%)	
4c	High/moderate suspicion	High/moderate suspicion for malignancy (50%–94%)	
5	Highly suggestive of malignancy	High probability of malignancy (≥95%)	Appropriate action should be taken/biopsy is recommended
6	Known biopsy-proven malignancy	Confirmed malignancy	Appropriate action should be taken

Specifications about mammography are marked in bold and specification about MBI are underlined.

MBI was performed 5%–10 minutes after intravenous injection of respectively 600 MBq (16.2 mCi) or 300 MBq (8.1 mCi) Tc-99m sestamibi using the single head Dilon 6800 gamma camera (Dilon Technologies, Newport News, VA, from April 2012 until December 2018), or the double head GE Discovery NM750b (SmartBreast, CA, from January 2019). Images were acquired in seated position, with light compression of the breast. Subsequent images were acquired in the craniocaudal (CC) and latero-oblique (LO) direction of both breasts and additional lateromedial and/or mediolateral view of the breast with nipple discharge. Occasionally, an additional axillary craniocaudal view was acquired if deemed necessary. All images were interpreted according to the lexicon for interpreting MBI^30^ (Table 1).

In case of suspicious breast lesions, fine needle aspiration cytology or core needle biopsy﻿ was performed for histopathologic analysis. If lesions could not be depicted with targeted US, MBI-guided biopsy (using GammaLoc, Dilon Technologies Inc., Newport News, VA) was performed whenever possible.^31^

### Outcomes

The primary outcome was the diagnostic accuracy (i.e., sensitivity, specificity, negative predictive value (﻿NPV), and positive predictive value (﻿PPV)) of MBI for the detection of malignancy in patients presenting with PND. For analytic purposes, BIRADS categories 1–3 were considered benign and categories 4–5 malignant. Histopathology was considered the gold standard. When no biopsy was performed, the patient was kept in follow-up for at least 1 year.

## RESULTS

Between April 2012 and September 2020, 91 females (mean age 66 y; range 34–94 years) presented with PND. Considering that one patient presented PND in both breasts, and 4 patients were treated but relapsed again with PND, 5 additional analyses were included. In total, 96 cases of PND were suitable for analysis in this study.

The majority of the patients (85%) had no previous history of breast disease, while 7% had a history of BC, 3% had a known benign lesion (fibroadenoma or cyst) or a history of benign cause of nipple discharge (4%). The majority (80%) had no family history of breast diseases and, particularly, there was no known BRCA mutation (Table 2). Thirteen patients (13,5%) reported the use of oral anticoagulants. Eight of the 13 patients used platelet aggregation inhibitors, 4 used coumarins, and 1 patient used direct factor Xa inhibitor (Table 2).

**TABLE 2:** Patient and Imaging Characteristics

Parameter	n (total=96)
Patient characteristics	
Affected breast	
Left	52
Right	44
History of breast anomalies	14
Cancer on the same side as PND	3
Cancer on the opposite side of PND	4
Benign anomalies	3
Benign nipple discharge	4
Family history of breast disease/cancer	19
Use of oral anticoagulants	13
Factor Xa inhibitor	1
Coumarins	4
Platelet aggregation inhibitor	8
Imaging characteristics	
BIRADS classification CD	
Benign	84
Malignant	12
BIRADS classification MBI	
Benign	72
Malignant	24

CD indicates conventional diagnostic imaging with mammography and ultrasound; MBI, molecular breast imaging; PND, pathologic nipple discharge.

All 96 cases were studied with MG, US, and MBI. Overall, CD imaging classified 12 cases as malignant and 84 as benign, while MBI classified 24 cases as malignant and 72 as benign (Table 2). The median time interval between CD imaging and MBI was 33 days, ranging from 0 to 209 days. In 80% of the patients, the time interval was less than 3 months (median of 12 days﻿ and mean of 20 days﻿).

**FIGURE 2:**
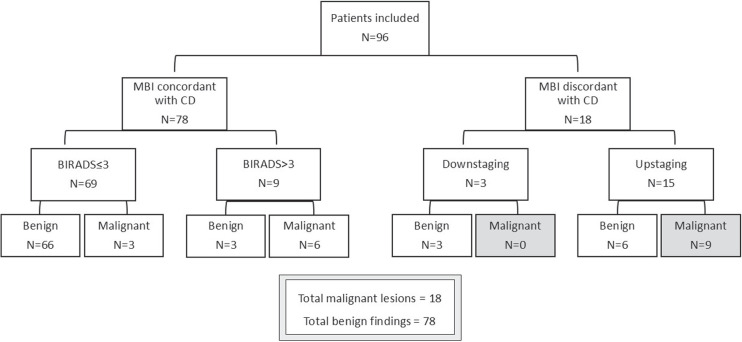
Schematic representation of the concordance between imaging findings and final diagnosis. CD indicates conventional diagnostic imaging with mammography and ultrasound.

The majority, 81% (78/96), of diagnostic images were concordant in their scores, either in classifying lesions as BIRADS≤3 in 69 cases or BIRADS>3 in 9 cases. In this group, there were 6 cases with incorrect findings, 3 BIRADS≤3 cases corresponded to breast cancer on biopsy and 3 BIRADS>3 cases corresponded to non-malignant entities (Fig. 2). Discordant findings between MBI and CD imaging were found in 19% of cases. From these, 83% (15/18) were upstaged by MBI, meaning they were classified as malignant. Nine out of 15 were confirmed to be malignant on biopsy. Three cases were all correctly downstaged by MBI (Fig. 2).

**FIGURE 3:**
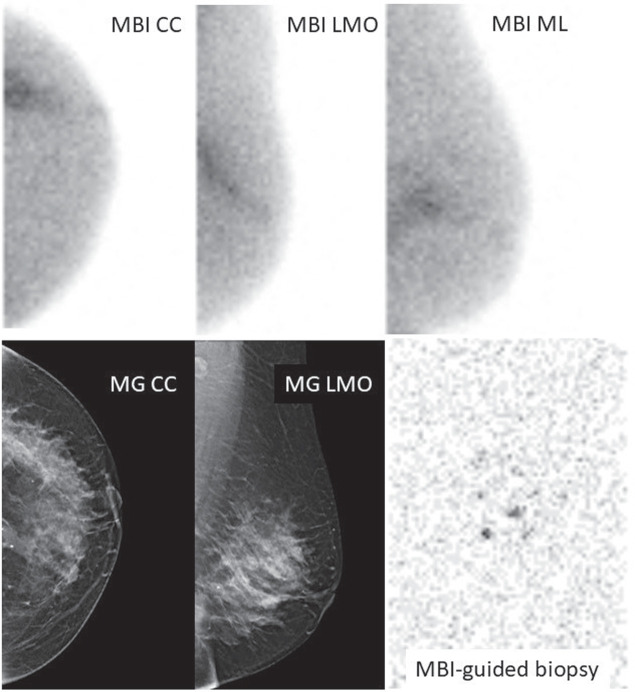
Illustration of a deep breast cancer lesion occult on CD but diagnosed after MBI-guided biopsy. Seventy-four-year-old woman with unilateral nipple discharge from the left breast. MG/US revealed an area of breast duct ectasia and cysts in the retroareolar region but no suspicion of malignancy (BIRADS 2). MBI performed 15 days after MG/US, showed increased uptake in the upper lateral quadrant of the left breast in a segmental pattern, measuring 7 cm, suspicious of malignancy (BIRADS 4). MBI-guided biopsy was compatible with a DCIS G2, and surgery revealed multifocal ADC NST in large area of DCIS G2. CC indicates craniocaudal; ML, mediolateral; MLO, mediolateral oblique.

**FIGURE 4:**
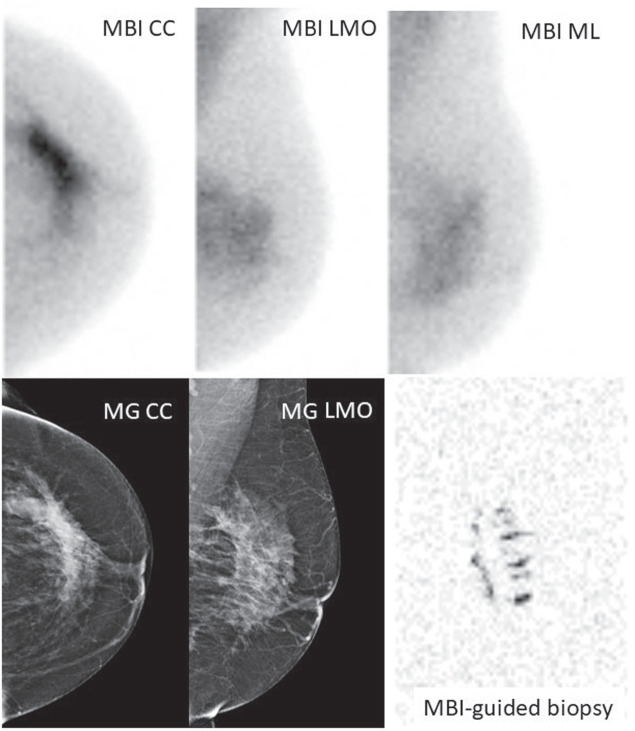
Illustration of the benefits of MBI-guided biopsy in case of discordant findings between CD and US-guided biopsy. Seventy-two-year-old woman with left nipple retraction and unilateral nipple discharge. MG/US showed asymmetry in the left upper lateral quadrant, suspicious of malignancy (BIRADS 4). US-guided biopsy revealed benign cells (sample error?). MBI performed 5 days after MG/US described a large area of pathological uptake affecting all quadrants, in an extension of 7 cm. MBI-guided biopsy was compatible with DCIS G2, which was confirmed after surgery. CC indicates craniocaudal; ML, mediolateral; MLO, mediolateral oblique.

**FIGURE 5:**
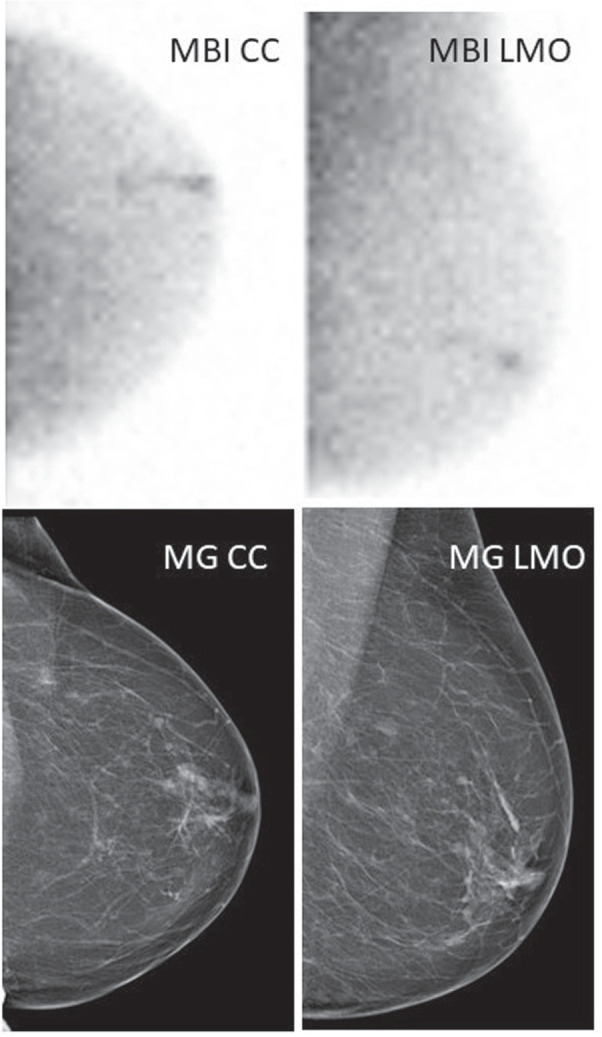
Illustration of a retroareolar breast lesion, occult on CD, but identifiable on MBI. Seventy-six-year-old woman with unilateral nipple discharge from the left breast. MG/US revealed duct ectasia and cysts in the retroareolar area of the left breast but no suspicion of malignancy (BIRADS 2). MBI performed 8 days after MG/US showed a linearly increased uptake behind the left nipple measuring 3.5 cm, suspicious of malignancy (BIRADS 4). Two days later, the substrate was found on the second look US. The US-guided biopsy revealed DCIS G3, which was also confirmed with surgery. CC indicates craniocaudal; ML, mediolateral; MLO, mediolateral oblique.

In total, 18 cases corresponded to breast cancer on biopsy, therefore, 20% (18/96) of all cases were malignant (Table 3). From these, 9 were detected by MBI only (Fig. 3), 6 were identified by both CD and MBI (Fig. 4) and 3 were false negative on both CD and MBI (BIRADS≤3). Although biopsy revealed ductal carcinoma in situ (DCIS) in the majority (78%) of cases, final histopathology after surgery revealed invasive cancer (adenocarcinoma no special type—ADC NST) in 44% and DCIS in 50% of the cases. The 18 histopathologically confirmed BC cases presented with a median size of 31 mm, ranging from 9 to 84 mm (Table 3). Furthermore, 2 cancers were located at <2.5 cm distance from the nipple (Fig. 5).

**TABLE 3:** Main Characteristics of the Breast Cancer Lesions

		Biopsy result		Surgery result			
Imaging modalities	Distance to the nipple on MBI	Subtype	Grade	Size	Subtype	Grade	Total (n=18)
Suspicious on both CD+MBI	>2.5 cm in 6	6 DCIS	2 G1 1 G2 1 G1/2 1 G3 1 NA	35 mm (10–70)	3 DCIS 3 DCIS+ADC NST	6 G2	6
Suspicious on MBI only	>2.5 cm in 7 ≤2.5 cm in 2	6 DCIS 1DCIS+ADC 1 ADC NST 1 NA	1 G1 6 G2 2 NA	27 mm (9–84)	5 DCIS 2 DCIS+ADC NST 2 ADC NST	5 G2 1 G3 3 NA	9
Non-suspicious on both CD+MBI (False negative)	>2.5 cm in 2 NA in 1	3 DCIS	1 G1 2 G2 1 NA*	21 mm 39 mm 1 NA*	1 ADC NST 1 DCIS 1 NA*	1 G2 1 G3 1NA*	3

* Despite DCIS on biopsy, the surgery only revealed mastopathy with no cancer cells.

ADC NST indicates adenocarcinoma no special type; CD, conventional imaging diagnosis; DCIS, ductal carcinoma in situ; G1, well differentiated; G2, moderately differentiated; G3, undifferentiated; MBI, molecular breast imaging; NA, not applicable/not possible to determine.

The great majority of cases, 81% (78/96), corresponded to benign findings (7 were confirmed by biopsy, the remaining during 1-year follow-up). From these, 85% (66/78) had a benign finding on all imaging modalities, 12% (9/78) presented a disagreement between CD and MBI BIRADS classification (6 were classified as BIRADS≤ 3 on CD, but BIRADS>3 on MBI, and 3 were classified as BIRADS>3 on CD, but BIRADS≤ 3 on MBI), and 4% (3/78) corresponded to false positive results in all imaging modalities (BIRADS >3) (Fig. 2).

When evaluating the agreement and disagreement between MBI and MG/US, the majority 81% (78/96) of cases received the same BIRADS classification, and, from these, 75% (72/96) were considered true positives or true negatives after biopsy or follow-up (Fig. 2). Differences in BIRADS classification between imaging modalities were found in 19% (18/96) of cases, one half corresponded to malignant lesions and the other half to benign lesions (Fig. 2). One should note that all 9 malignant cases with discordant BIRADS classification, were classified as malignant by MBI (BIRADS >3), but had BIRADS ≤3 on CD. Therefore, in this specific context of disagreement in BIRADS classification, MBI presented 100% NPV.

MBI presented a sensitivity of 83%, specificity of 89%, PPV of 63%, and NPV of 96% (Table 4). CD alone showed a sensitivity of 33%, specificity of 92%, PPV of 50%, and NPV of 86% (Table 4). The receiver operating curve (ROC) analysis showed good diagnostic accuracy of MBI to detect malignant lesions with an AUC of 0.86 [*P*-value<0.001 (95% CI: 0.75–0.97)]. CD alone presented fair diagnostic accuracy with an AUC of 0.63 [*P*-value=0.091 (95% CI: 0.47–0.79)].

**TABLE 4:** Diagnostic Accuracy of MBI and CD

	Malignant lesion	Benign lesion	Sensitivity (%)	Specificity (%)	PPV (%)	NPV (%)
MBI						
Malignant (BIRADS>3)	15	9	83	89	63	96
Benign (BIRADS≤3)	3	69	—	—	—	—
CD						
Malignant (BIRADS>3)	6	6	33	92	50	86
Benign (BIRADS≤3)	12	72	—	—	—	—

NPV indicates negative predictive value; PPV, positive predictive value.

## DISCUSSION

In this retrospective cohort single-center study, our main goal was to evaluate the performance of MBI with Tc-99m sestamibi to rule out malignancy in women with clinically suspicious PND and negative or discrepant findings on CD. Of the 96 included cases, 18 corresponded to breast cancer, and 78 to benign etiology.

The incidence of breast cancer in our study population was 20%, which resembles the incidence mentioned in the literature (up to 16%). However, the incidence in the whole group of patients presenting with PND is probably higher, since PND cases with clear breast cancer on CD did not undergo MBI in our institution and were not included in this study. This might have led to selection bias.

In our study, adjunct MBI showed higher sensitivity (83% vs. 33%) and NPV (96% vs. 86%) than CD alone, with similar specificity (89% vs. 92%) and PPV (63% vs. 50%), this may be enhanced by the highly selected population in which patients with BIRADS>3 on CD were excluded. Despite the ROC curves not being very accurate since based on a single sensitivity/specificity combination, MBI demonstrated a better performance when compared with CD alone, presenting a good diagnostic accuracy to detect malignant lesions with a statistically significant AUC of 0,86 (95% CI: 0.75–0.97), compared with CD alone with AUC of 0.63 (95% CI: 0.47–0,79). Of note, 50% of all malignant lesions (9/18) were depicted by MBI only, and fewer malignancies were missed by MBI compared with CD (3 vs. 12). Moreover, based on MBI results of the cases with discordant BIRADS classification that were upstaged (15/96—Fig. 2), the additional risk of a possible futile biopsy by adding MBI in the diagnostic workup seems to be very low due to its high specificity (6/96 false positives only).

To our knowledge, only one study has been published investigating MBI with Tc-99m sestamibi in patients with a history of PND. In this pilot study, including 14 patients, no malignant case was classified with MBI, and the authors concluded that the concordance between MBI, MG, and US regarding the absence of suspicious lesions in these patients was reassuring (NCT00566280).^32^ In our study, we found an agreement between the 3 imaging modalities in 78 of 96 cases (81%).

Although ruling out malignancy is essential in a patient with PND, the use of MBI in this specific clinical setting is not specifically addressed in the SNMMI Procedure Standard/EANM Practice Guideline for Molecular Breast Imaging with Dedicated gamma-Cameras published in 2022.^22^ Moreover, the American College of Radiology Appropriateness Criteria Evaluation of Nipple Discharge, updated in 2022, stated that there is no relevant literature to support the use of MBI in the evaluation of patients with PND.^9^ In this respect, taking our results into account, we believe that our study will fill a gap in the scientific literature, leading to improvement in the clinical management of patients with PND. In addition, MBI may help identify lesions located in unexpected regions, such as the deeper parts of the breast. The latter is particularly important in the context of PND, because conus excision may not be beneficial for the patient in such cases. Actually, in our patient cohort, MBI identified only 2/9 cancers occult on CD behind or <2.5 cm from the nipple, meaning that, without additional MBI, a significant number of patients might have undergone futile central duct excision.

Commonly, breast MRI is performed in cases in which MG and US have not identified the cause of PND. Its sensitivity for detecting the cause of the PND ranges between 86%–100% for invasive cancer and 40%-100% for noninvasive disease.^6,9,12,16,17,33–36^ Bahl et al^36^ retrospectively reviewed 91 women with PND and found that the sensitivity, specificity, PPV, and NPV of MRI for detecting malignancy was 100%, 68%, 37%, and 100%, respectively.^36^ However, the study population was not restricted to patients with PND only, because other features of PND, such as unilateral, spontaneous, or serous discharge were also included. Paradoxically, in the retrospective study from van Gelder et al^17^ evaluating a specific cohort of 111 women with unilateral bloody nipple discharge and without suspicious findings on both MG and US, malignancy was identified by MRI in only 2 patients (1,8%), both corresponding to DCIS. These authors concluded that MRI was not indicated as initial imaging to assess PND.

The need for radiopharmaceuticals to perform MBI, and the resulting radiation exposure is a commonly mentioned disadvantage. In our center, patients received an intravenous injection of 600 MBq Tc-99m sestamibi, which is equivalent to 5 mSV on the whole body, when using the single-detector camera but, with the installation of a double detector camera with more sensitive cadmium-zinc-telluride (CZT) crystals, the administered activity could be reduced by half (300 MBq). This value may be further reduced by using low-dose protocols, allowing for a significant reduction of the absorbed dose to the breast (1.1 mSv).^37,38^ In addition, the potential risk of cancer due to radiation exposure from MBI has been described as merely speculative and theoretical. Radiation-induced cancer caused by doses below 100 mSv are not statistically different from zero, and the United Nations Scientific Committee on Exposure to Atomic Radiation cautions against extrapolating “radiation-induced health effects within a population exposed to incremental doses at levels equivalent to or lower than natural background levels” of 2–10 mSv.^39,40^

Gadolinium-based contrast agents (GBCAs) available for MRI improve breast imaging performance. The incidence of acute adverse reactions to GBCAs is relatively low, approximately 3- to 5-fold less than that of iodinated contrast agents used for MG. However, late adverse effects have been reported, such as nephrogenic systemic fibrosis in patients with severe chronic or acute kidney failure.^41,42^ Another factor that has raised some concern is the GBCA retention in human tissues, including the brain.^41,42^ Moreover, GBCAs have been found in sewage water, surface water, and drinking water worldwide, raising environmental concerns.^43^ Future research regarding contrast dose reduction and noncontrast diffusion-weighted imaging-based protocols for breast MRI screening are being developed and look promising.

The main strengths of this study are its originality (since it is the second study aiming to evaluate the role of MBI with Tc-99m sestamibi in a specific population with PND), the homogenous cohort definition, the size of the sample (the other study about this topic included 14 patients only, while we included 96 cases which makes it the largest population evaluated with MBI until now), the systematic comparison between MG/US and MBI, the standardized reading using the Lexicon,^30^ and the pathologic confirmation and/or adequate follow-up as gold standard to confirm or exclude malignancy. The principal limitations are the fact of being a retrospective, single-centre study and data were included since 2012 (despite no major changes in the investigation procedure occurred during the data collection period of time).

Although MBI lacks large scale availability in many countries and it is not the first-line approach to study PND, it is important to emphasize its good diagnostic accuracy to evaluate clinically suspicious breast lesions that are undetermined or negative on CD. It is a good alternative to MRI as an adjunct imaging modality, that is read together with MG, especially when taking into account its advantages (eg, less time consuming, less expensive, fast learning curve, and may be more appropriate in specific patients such as the ones with implants, obese, or claustrophobic).

## CONCLUSIONS

The diagnostic workup of patients presenting PND and negative or inconclusive findings on CD is challenging. The addition of MRI remains debatable. The use of MBI as an adjunct modality in this patient population showed higher sensitivity and NPV compared with CD alone, leading to fewer missed malignancies and potentially reducing futile central duct excisions. This reinforces the role of MBI in the diagnostic algorithm of patients with PND.
